# Crystal structure of human S100A8 in complex with zinc and calcium

**DOI:** 10.1186/s12900-016-0058-4

**Published:** 2016-06-01

**Authors:** Haili Lin, Gregers Rom Andersen, Laure Yatime

**Affiliations:** Department of Molecular Biology and Genetics, Aarhus University, Gustav Wieds Vej 10C, DK-8000 Aarhus, Denmark; Present address: DIMNP – UMR5235, University of Montpellier, Place Eugène Bataillon, Bât. 24 cc107, 34095 Montpellier Cedex 5, France

**Keywords:** S100 proteins, EF-hand, Calcium, Zinc, Oligomerization

## Abstract

**Background:**

S100 proteins are a large family of calcium binding proteins present only in vertebrates. They function intra- and extracellularly both as regulators of homeostatic processes and as potent effectors during inflammation. Among these, S100A8 and S100A9 are two major constituents of neutrophils that can assemble into homodimers, heterodimers and higher oligomeric species, including fibrillary structures found in the ageing prostate. Each of these forms assumes specific functions and their formation is dependent on divalent cations, notably calcium and zinc. In particular, zinc appears as a major regulator of S100 protein function in a disease context. Despite this central role, no structural information on how zinc bind to S100A8/S100A9 and regulates their quaternary structure is yet available.

**Results:**

Here we report two crystallographic structures of calcium and zinc-loaded human S100A8. S100A8 binds two zinc ions per homodimer, through two symmetrical, all-His tetracoordination sites, revealing a classical His-Zn binding mode for the protein. Furthermore, the presence of a (Zn)_2_-cacodylate complex in our second crystal form induces ligand swapping within the canonical His_4_ zinc binding motif, thereby creating two new Zn-sites, one of which involves residues from symmetry-related molecules. Finally, we describe the calcium-induced S100A8 tetramer and reveal how zinc stabilizes this tetramer by tightening the dimer-dimer interface.

**Conclusions:**

Our structures of Zn^2+^/Ca^2+^-bound hS100A8 demonstrate that S100A8 is a genuine His-Zn S100 protein. Furthermore, they show how zinc stabilizes S100A8 tetramerization and potentially mediates the formation of novel interdimer interactions. We propose that these zinc-mediated interactions may serve as a basis for the generation of larger oligomers in vivo.

**Electronic supplementary material:**

The online version of this article (doi:10.1186/s12900-016-0058-4) contains supplementary material, which is available to authorized users.

## Background

S100 proteins belong to the EF-hand calcium-binding protein superfamily and count more than 20 members that are expressed exclusively in vertebrates, in a tissue- and cell-specific manner [1, 2]. Under homeostatic conditions, they are found in the cytoplasm or in the nucleus of eukaryotic cells where they regulate vital processes, generally in a calcium-dependent manner [1, 2]. S100 protein expression is often upregulated during inflammation. In addition, these proteins can be secreted or passively released in the extracellular matrix where they become damage-associated molecular patterns (DAMPs) and exert cytokine-like functions, through specific membrane-bound receptors [3–6], thus promoting sustained inflammation and tissue damage in various pathological settings associated with cardiovascular complications, neurodegenerative disorders and cancers [1–3, 7]. As a consequence, S100 proteins are considered both as valuable biological markers and important therapeutical targets for various inflammatory conditions [8, 9].

S100A8 and S100A9 constitute two of the most potent pro-inflammatory molecules among the S100 protein family [10]. They are constitutively expressed by leukocytes of myeloid lineage [11, 12]. Although products of distinct genes, the two proteins are often co-expressed and account for up to 45 % of the total cytosolic protein pool in human neutrophils [13]. As for all their congeners, S100A8 and S100A9 form homodimers as a minimal, active unit [14, 15]. However, they usually exert their physiological function as a S100A8/A9 heterodimer, also termed calprotectin, and they can further associate via heterotetramerization [16, 17]. The S100A8/A9 heterodimer is proposed to regulate various homeostatic processes including fatty acid transport, cytoskeleton reorganization and myeloid cell differentiation [18–20]. S100A8 and S100A9 have no signal sequences for classical secretion, but can be released from activated leukocytes, either by active secretion via a tubulin-dependent pathway, or by passive release, within neutrophil extracellular traps (NETs) or from necrotic cells [21–23].

As for all S100 proteins, S100A8, S100A9, and their heterodimer bind two calcium ions per monomer, one in each EF-hand motif [14, 15, 17]. In addition to calcium, calprotectin can bind Zn^2+^, Mn^2+^, Cu^2+^, and Fe^2+^ and, by efficiently sequestrating these divalent cations in the extracellular environment, it acts as a potent antimicrobial agent [24–28]. Interestingly, this antibacterial property is restricted to the S100A8/A9 heterodimer [29] although both homodimers are expected to bind zinc as well, based on sequence comparisons [30]. Indeed, zinc binding has been reported for several members of the S100 protein family [30] and the determination of the three-dimensional structures of both S100B, S100A7, S100A12 and S100A15 complexed to zinc has revealed two common, symmetrical zinc binding sites at the dimer interface formed by highly conserved histidines and possibly one or two aspartate/glutamate [31–34]. These Zn^2+^-coordinating residues are conserved in S100A8 and S100A9, suggesting that both proteins could bind zinc in a similar fashion. However, no structural data are yet available to precisely characterize the zinc binding mode of either S100A8 and S100A9 homodimers or S100A8/A9 heterodimer. Zinc was furthermore shown to enhance the oligomerization of these proteins, either as homo- and heterotetramers or as much more complex amyloid structures [35, 36]. Finally, it has been proposed that the activity of extracellular S100 proteins, including S100A8 and S100A9, would be regulated by other divalent cations than calcium, notably Zn^2+^, since the two EF-hand Ca^2+^-binding sites would be fully saturated at all time in the extracellular compartment due to the high calcium concentrations [30]. Zinc appears therefore as an important regulator of the structural organization and the resulting functional properties of both S100A8 and S100A9. However, no structural information is yet available to decipher the intimate mechanisms by which this regulation is achieved.

To better understand how zinc modulates the structure-function relationship of S100A8 and S100A9, we have undertaken the structural studies of these S100 proteins in the presence of zinc. Here we report the crystallographic structures of human S100A8 (hS100A8) bound to zinc and calcium at 2.2 and 2.1 Å, respectively. The first structure reveals a classical, all-histidine zinc-binding motif, thereby classifying S100A8 as a genuine His-Zn S100 protein. The second structure displays an atypical (Zn)_2_-cacodylate complex in one of the two Zn-sites per homodimer, leading to ligand swapping and creation of two novel Zn-sites. Analysis of the quaternary arrangement of hS100A8 in both crystal forms suggests that zinc enhances tetramer stabilization and could further participate in the formation of higher oligomeric species and/or in the interaction with S100A8 binding partners.

## Results and discussion

### A classical His-Zn binding motif

**Fig. 1:**
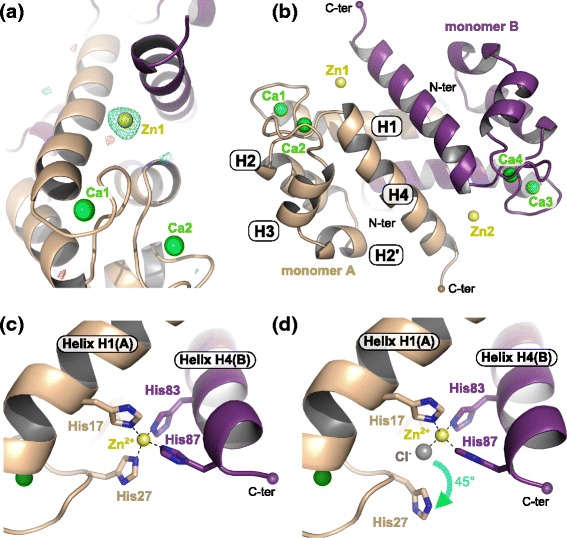
The crystallographic structure of Zn^2+^/Ca^2+^-bound hS100A8. **a** Anomalous difference Fourier maps calculated using phases and weight from the best refined atomic model (without ions) obtained with the native dataset (crystal form 1) and anomalous differences from the datasets collected at wavelengths of 1.27 Å (cyan mesh, contour at 3.5 σ) and 1.30 Å (red mesh, contour at 3.5 σ). The final model displayed as cartoon in beige and purple is superimposed for comparison. The anomalous signal clearly disappears for the wavelength above the Zn maximal absorbance peak (λ = 1.30 Å) as judged by the fact that almost no density is visible for the second anomalous difference Fourier map (red mesh). **b** Overview of the structure of Zn^2+^/Ca^2+^-bound hS100A8 derived from crystal form 1 at 2.2 Å resolution showing two zinc bound at the homodimer interface (yellow spheres). The four calcium ions are displayed as green spheres. **c** Close-up view on the Zn^2+^-binding site revealing an all-His binding motif formed by two residues from each monomer. **d** In two out of eight molecules within the asymmetric unit, His27 from monomer A is replaced by a chloride ion (gray sphere)

In the presence of both zinc and calcium, hS100A8 gave rise to two new crystal forms displaying a P2_1_2_1_2_1_ and a C222_1_ symmetry, as compared to Ca^2+^-bound hS100A8 which crystallized in P3_1_21 [14] (Table 1). Both structures could nevertheless be solved by molecular replacement using the previously reported Ca^2+^-hS100A8 homodimer structure. For crystal form 2 (C222_1_), structure determination was also successful using SAD-phasing in PHENIX.AUTOSOLVE [37] from the dataset collected at the Zn peak (λ = 1.27 Å) (Additional file 1: Table S1). In that case, 12 sites were identified corresponding to the eight Zn^2+^ ions and four of the eight Ca^2+^ ions (Additional file 2: Figure S1a). The overall figures of merit after SAD-phasing and after density modification using RESOLVE were 0.36 and 0.73, respectively (35–2.1 Å resolution cut-off). In any case, the position of the zinc ions could be unambiguously identified in both structures from anomalous difference Fourier maps calculated using phases and figure of merit weights from the best refined atomic model containing no ions obtained with the native dataset and anomalous differences from the datasets collected at wavelengths of 1.27 and 1.30 Å (Additional file 1: Table S1, Fig. 1a and Additional file 2: Figure S1b-c).

**Table 1:** Data collection and refinement statistics

	Crystal form 1	Crystal form 2
Data collection		
Diffraction source	I911-3, MAX-lab	I911-3, MAX-lab
Wavelength (Å)	1.0	1.0
Space group	P2_1_2_1_2_1_	C222_1_
*a*, *b*, *c* (Å)	50.93, 85.09, 197.20	55.98, 90.03, 196.80
α, β, γ (°)	90, 90, 90	90, 90, 90
Mosaicity (°)	0.14	0.25
Resolution range (Å)	50–2.2 (2.3–2.2)	50–2.1 (2.2–2.1)
Total No. of reflections	262,642	232,345
No. of unique reflections	42,972	29,069
Completeness (%)	96.5 (99.6)	98.5 (97.8)
Redundancy	6.1 (6.0)	8.0 (8.2)
*I*/σ(*I)*	12.54 (3.08)	16.47 (3.04)
*R*_meas_	14.8 (78.8)	9.5 (86.4)
Wilson B factor (Å^2^)	30.6	37.1
Refinement		
Resolution range (Å)	40–2.2	49–2.1
No. reflections, working + test set	41,886	28,412
Final R_work_/R_free_ (%)	18.19/21.66	17.85/19.70
No. of non-H atoms		
Protein	5791	3009
Ions	26	24
Ligands	24	26
Water	358	193
Total	6199	3252
R.m.s. deviations		
Bonds (Å)	0.002	0.002
Angles (°)	0.439	0.521
Average B factors (Å^2^)		
Protein	33.9	43.9
Ions	32.1	42.7
Ligands	44.9	76.1
Water	37.0	47.7
Ramachandran plot		
Favored regions (%)	99.4	99.7
Additionally allowed (%)	0.6	0.3
Outliers (%)	0	0

Values for the outer shell are given in parentheses

The overall structure of Zn^2+^/Ca^2+^-bound hS100A8 from crystal form 1 is displayed in Fig. 1b. The protein arranges in a canonical homodimer that contains four calcium ions, one in each EF-hand, and two zinc ions at the interface between the two monomers (Fig. 1b). Although zinc can replace calcium in the EF-hand motifs when present alone [35], only Ca^2+^ was found in all EF-hands of our model as judged from the anomalous difference Fourier maps (Fig. 1a). The two symmetrical Zn-sites are formed by two residues from each monomer: from the first monomer, His17 in helix H1 and His27 in the first EF-hand, and from the second monomer, His83 and His87 at the C-terminus of helix H4 (Fig. 1c). His27 appears however somewhat flexible and in two out of the eight molecules present in the asymmetric unit of our structure, it is flipped away from the Zn-site by around 45°, the missing coordination being instead provided by a chloride ion (Fig. 1d). In any case, the Zn-binding mode of hS100A8 is very similar to that of the four other S100 proteins for which crystal structures in the presence of zinc have been reported, namely S100A7, S100A12, S100A15, and S100B [31–34] (Additional file 3: Figure S2), with the particularity that S100A8 is the sole among these S100 proteins to display an all-histidine zinc binding site. Our structural data therefore clearly confirm that hS100A8 belongs to the His-Zn group of S100 proteins.

### An atypical Zn-site formed by a (Zn)_2_-cacodylate complex

**Fig. 2:**
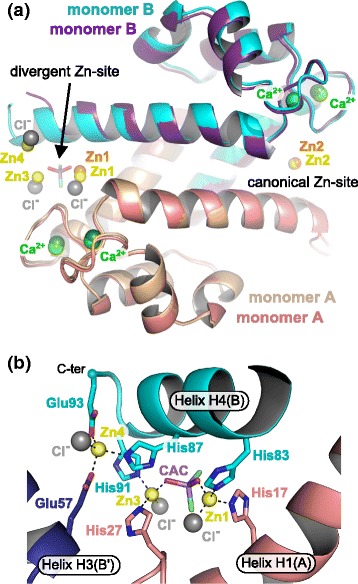
The atypical (Zn)_2_-cacodylate complex present in crystal form 2 induces the formation of new Zn-sites. **a** Superimposition of the hS100A8 homodimers from crystal form 1 (beige and purple with Zn^2+^ ions in orange) and crystal form 2 (cyan and salmon with Zn^2+^ ions in yellow), respectively. Only one Zn-site (Zn2) is conserved between the two structures. **b** Close-up view on the new Zn-sites observed in the structure derived from crystal from 2. The presence of the (Zn)_2_-cacodylate complex induces ligand swapping and the formation of two novel Zn-sites (Zn3 and Zn4)

Surprisingly, in the structure derived from crystal form 2, four zinc ions per homodimer were present in addition to the four calcium ions (Fig. 2a). A closer inspection of the structure revealed that only one of the two symmetrical Zn-sites found in crystal form 1 was strictly conserved (Zn2) whereas the second site (Zn1) was instead replaced by a (Zn)_2_-cacodylate complex, the cacodylate molecule coming from the crystallization condition (Fig. 2b). In this new site, Zn1 (crystal form 2) occupies almost the same position as Zn1 from crystal form 1 and is coordinated by His17 from monomer A and His83 from monomer B. The two other coordinating His residues are however replaced by a chloride ion and by one of the hydroxyl groups of the cacodylate molecule. Instead, His27 from monomer A and His87 from monomer B are flipped by 70 and 100°, respectively. As a consequence, His27 now coordinates the second Zn^2+^ (Zn3) from the (Zn)_2_-cacodylate complex which is further held in place by His91 from monomer B, by a second chloride ion and by the second hydroxyl group of the cacodylate molecule. Finally, the new position of His87 leads to the creation of a third Zn-site (Zn4) which is completed by Glu93 from the very C-terminal end of helix H4 (monomer B), Glu57 from a symmetry-related molecule, and a third chloride ion (Fig. 2b). Despite this quite large rearrangement of one of the Zn-sites in crystal form 2, the resulting S100A8 homodimer structure remains unaffected and superimposes well with the homodimer from crystal form 1, with an overall root-mean-square deviation (r.m.s.d.) on Cα atoms of 0.464 Å. Hence it seems possible to accommodate more than just one Zn^2+^ ion at the dimer interface within S100A8, and, in addition to the four conserved histidines from the canonical Zn-binding motif, other residues such as His91 or Glu93 can participate in zinc coordination. Whether such additional Zn^2+^ sites occur *in vivo* and what their functional relevance might be still remains to be determined.

### Influence of divalent cations on S100A8 oligomerization

**Fig. 3:**
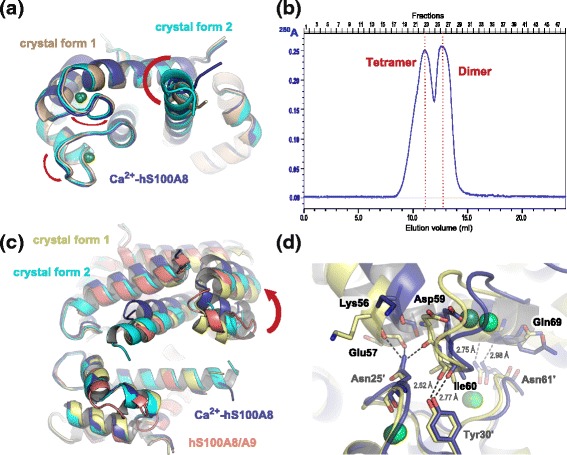
Oligomeric states of hS100A8 in the presence of divalent cations. **a** Superimposition of the Zn^2+^/Ca^2+^-bound hS100A8 homodimers from crystal form 1 (beige) and crystal form 2 (cyan) with Ca^2+^-hS100A8 (dark blue) [14]. The major regions of divergence are indicated with red arcs. **b** Elution profile of hS100A8 on a 24 ml Superdex 75 size exclusion chromatography column (Ge Healthcare Life Sciences) equilibrated in 20 mM HEPES pH 7.5, 200 mM NaCl, 5 mM CaCl_2_. Ca^2+^-hS100A8 elutes as two peaks with elution volumes of 11.15 and 12.8 ml, respectively, most likely corresponding to a tetramer and a dimer, respectively. **c** Superimposition of the Zn^2+^/Ca^2+^-bound hS100A8 homotetramers from crystal form 1 (yellow) and crystal form 2 (cyan) with the homotetramer derived from the crystal packing in Ca^2+^-hS100A8 (dark blue, [14]) and with the (S100A8/S100A9)_2_ heterotetramer (salmon, [17]). The red arrow indicates the movement of one S100A8 homodimer away from the other homodimer on one side of the dimer-dimer interface in the calcium-loaded hS100A8 [14]. **d** Close-up view on the interactions stabilizing the homodimer-homodimer packing within the hS100A8 homotetramer from Ca^2+^-hS100A8 (purple) and from Zn^2+^/Ca^2+^-hS100A8 (yellow, crystal from 1)

The Zn^2+^/Ca^2+^-hS100A8 homodimer we observe is highly similar to the previously reported Ca^2+^-hS100A8 homodimer [14] with an r.m.s.d. on Cα atoms of 0.65 and 0.45 Å between the Ca^2+^-loaded homodimer and the Zn^2+^/Ca^2+^-bound homodimers from crystal forms 1 and 2, respectively (Fig. 3a). The most prominent changes are observed for the C-terminal part of helix H4, which even forms an additional helix turn in the model derived from crystal form 2. In crystal form 1, helix H4 is not involved in crystal packing. In crystal form 2, helix H4 only interacts with Glu57 from a symmetry-related molecule due to the presence of the (Zn)_2_-cacodylate complex. The movement observed for helix H4 in both crystal forms is therefore more likely due to the presence of the Zn sites rather than to a different crystal packing as compared to Ca^2+^-hS100A8. Minor changes, mostly side chain repositioning of residues near the zinc binding pocket, are also noted around the two calcium EF-hands. Zinc binding was reported to increase both calcium affinity and target recognition for several S100 proteins, including S100B and S100A12 [30, 38, 39]. Based on structural data, it was proposed that increased target recognition is due to the repositioning of helix H4 C-terminus, which is believed to delineate the substrate binding cleft for many S100 proteins, whereas higher affinity for calcium is due to the stabilization of the Ca^2+^-bound EF-hands active conformation [32, 34], although no major conformational changes are observed around the EF-hands between the Ca^2+^-bound form and the Zn^2+^/Ca^2+^-bound form of both S100B and S100A12. In our crystal structures of Zn^2+^/Ca^2+^-hS100A8, the two calcium EF-hands superimpose well with the EF-hands of Ca^2+^-hS100A8 [32], as shown in Fig. 3a, and no major side chain repositioning is observed except for the residues involved in zinc coordination. However, a closer inspection of the Ca-O distances between the calcium ions and their coordinating residues present in the EF-hands of both Ca^2+^-hS100A8 and our two Zn^2+^/Ca^2+^-hS100A8 structures reveals that several of these Ca-O distances are significantly shortened upon Zn-binding (Table 2). Such direct comparison is possible since all three structures were solved at similar resolutions around 2 Å. Thus calcium binding by hS100A8 seems to be tighter in the presence of zinc. These observations taken together with the fact that the changes observed in hS100A8 upon zinc binding are very similar to those described for S100B and S100A12 suggest that calcium affinity could be enhanced by Zn^2+^ for hS100A8. As we do not know where interacting partners would bind on hS100A8, we cannot conclude whether Zn^2+^ could also have an effect on target recognition for S100A8 or not.

**Table 2:**
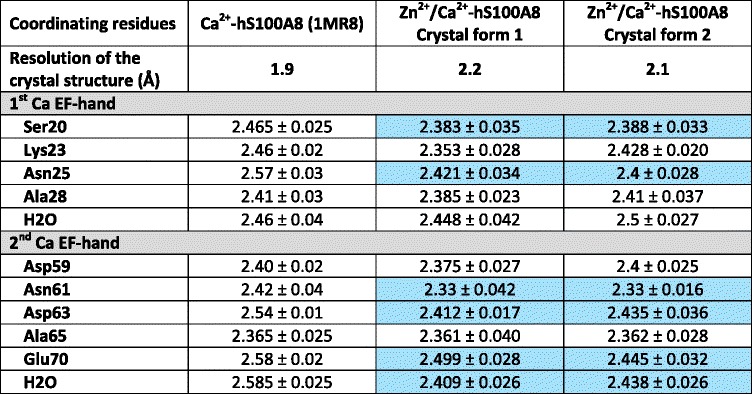
Average Ca-O distances in the two calcium EF-hands of the different hS1008 structures in Å

The Ca-O distances for which a significant change is observed upon Zn^2+^ presence are highlighted in blue

Oligomerization of S100 proteins strongly relies on divalent cations [40]. As for many other S100 proteins, S100A8 and S100A9 can readily form homo- and heterodimers in the absence of calcium [35] but the formation of the (S100A8/S100A9)_2_ heterotetramer is strictly Ca^2+^-dependent [17, 35]. Since the (S100A8/S100A9)_2_ heterotetramer is the most abundant form for the two proteins *in vivo*, most studies to evaluate the influence of divalent cations on S100A8/A9 have been performed on this oligomer. Thus little is known about the behaviour of S100A8 and/or S100A9 homo-oligomers in the presence of zinc, despite the fact that these forms have functions of their own [41, 42] that might also be regulated by divalent cations. In the sole presence of calcium, we observe that hS100A8 elutes as two separate oligomeric forms on a size exclusion chromatography (SEC) column (Fig. 3b). Based on the calibration curve of the SEC column (Additional file 4: Figure S3), we calculated that these two forms respectively encompass 2.5 (peak 1) and 1.25 (peak 2) S100A8 homodimers, and we therefore assigned them as homotetramer and homodimer, respectively. Thus, S100A8 homodimers can further associate into homotetramers during SEC. This tetrameric form was already noticeable in the crystal packing of the Ca^2+^-hS100A8 structure [14] although the authors did not comment on it. We observe a similar tetramer in our two distinct crystal forms (Fig. 3c) and the total buried surface area at the dimer-dimer interface is 1024 Å^2^ per dimer according to PISA [43]. Interestingly, the same tetrameric arrangement is observed for the (S100A8/S100A9)_2_ heterotetramer [17, 28] and all four tetramers superimpose quite well (Fig. 3c) suggesting that this tetramer is not dependent on very specific crystallization conditions and therefore may correspond to the tetramer observed in our size exclusion chromatography experiment. Within the hS100A8 homotetramer, the two homodimers are held in place by two symmetrical interfaces formed by the insertion of the second EF-hand from monomer B into the cleft between the two EF-hands of monomer A from the opposite homodimer (Fig. 3d). In particular, the carbonyl groups of the main chain of Lys56, Glu57, Asp59 and Ile60 (monomer B) interact with the side chains of either Asn25 or Tyr30 (1^st^ EF-hand, monomer A) while Gln69 carbonyl group (monomer B) connects with the side chain of Asn61 (2^nd^ EF-hand, monomer A). Interestingly, in Ca^2+^-hS100A8, the second hS100A8 homodimer is pulled away from the interface on one side, due to the slightly different orientation of helix H4 (Fig. 3c). As a consequence, one of the two homodimer-homodimer interfaces is strongly destabilized due to the breaking of the hydrogen bonds made by Asn25 side chain and the lengthening of all the other interactions described above (Fig. 3d). These observations suggest that zinc further enhances stabilization of the S100A8 tetramer.

## Conclusions

Here we report the crystal structure of human S100A8 in the presence of both calcium and zinc. The structure reveals an all-histidine Zn-binding site and demonstrates that hS100A8 belongs to the His-Zn group of S100 proteins, as expected from sequence comparisons. The structure further reveals that zinc allows tightening of the dimer-dimer interface within hS100A8 homotetramer, thus pointing towards an enhancing role of zinc in S100A8 tetramerization. Our data therefore suggest that, as for several other S100 proteins, divalent cations, and notably zinc, influence S100A8 oligomerization. The second Zn^2+^/Ca^2+^-hS100A8 structure presented here describes how an atypical (Zn)_2_-cacodylate complex can insert into one of the canonical Zn-sites and disrupt the classical His_4_ tetracoordination sphere, inducing ligand swapping and the formation of two new Zn-sites. This is not unique to S100A8 as a pH-dependent ligand swapping for zinc coordination has also been described for S100B [32]. Furthermore, both S100A8 and S100A9 contains several histidine residues on their C-termini and they can all be involved in the coordination of divalent cations as seen in our S100A8 structures or in the structures of Mn^2+^-bound calprotectin [28, 44]. Taken together with our structural data, these observations suggest that the histidine-rich C-termini of both S100A8 and S100A9 are quite flexible and prone to ligand swapping in the presence of divalent cations. Reorganization of the canonical Zn-sites leading to the involvement of new Zn-coordinating residues and/or the creation of new Zn-sites, possibly at the interface between two S100 dimers or between S100A8 and a binding partner, is a plausible scenario that could also occur *in vivo* under specific conditions. Ligand swapping in these flexible C-terminal regions could therefore potentially contribute to the formation of novel interdimer interactions, through ions coordination, that would lead to the formation of higher oligomeric species. Similarly, reorganization of the flexible C-terminus of both S100A8 and S100A9 by ligand swapping could induce the formation of cation binding sites at the interface between these S100 proteins and specific binding targets, the metal coordination being provided by both interacting partners. Such mechanisms would provide a structural rationale for the influence of zinc on S100:target recognition, as well as on the formation of S100A8/A9 amyloid-like structures which is known to be regulated by both calcium and zinc [36, 40].

## Methods

### Cloning, expression and purification of human S100A8

The gene coding for hS100A8, codon-optimized for bacterial expression, was purchased from Genscript (GenScript USA Inc.) and recloned into the Nco I – Hind III fragment of vector pETM13 (EMBL vector collection) to allow expression of the protein without any tags. To be in frame with the start codon in pETM13, two additional nucleotides (C + A) were introduced in between the Nco I site and the beginning of the hS100A8 sequence, yielding a protein with an additional alanine residue in between the initial methionine and the following leucine residue.

hS100A8 was expressed in *Escherichia coli* BL21(DE3) cells and purified in a three-step procedure including affinity chromatography on a 5 ml HisTrap FF Ni-column (GE Healthcare Life Sciences), anion exchange on a 9 ml Source 15Q column (GE Healthcare Life Sciences) and size exclusion chromatography (SEC) on a 120 ml home-packed Superdex 75 column. BL21(DE3) cells transformed with the hS100A8-pETM13 plasmid were grown at 310 K until their absorbance at 600 nm reached 0.6–0.8. Protein expression was then induced overnight at 291 K by addition of 1 mM IPTG. The cells were harvested by centrifugation, resuspended in a buffer containing 50 mM HEPES pH 7.5, 200 mM NaCl, 30 mM imidazole, 1 mM PMSF (Buffer A) and disrupted by sonication. After clarification by centrifugation, the supernatant was loaded onto the 5 ml Ni-column equilibrated in Buffer A and the non-specifically bound contaminants were washed off with high salt (Buffer A containing 1 M NaCl). As hS100A8 contains several histidine residues on its C-terminus, it binds relatively strongly to the Ni-column, even in the absence of a His-tag, and could be eluted with a buffer consisting of 50 mM HEPES pH 7.5, 200 mM NaCl, 250 mM imidazole, 1 mM PMSF. The protein was then dialyzed overnight at 277 K against 50 mM Tris-HCl pH 8.8, 50 mM NaCl and loaded onto the anion-exchange column. Elution was performed with a 100 ml gradient from 50 to 300 mM NaCl. hS100A8 appeared in the run-through fraction and in the first fractions of the gradient while most of the contaminants bound more strongly to the Source 15Q column. The hS100A8-containing fractions were pooled and concentrated to 5 ml before loading onto the SEC column equilibrated in 20 mM HEPES pH 7.5, 100 mM NaCl, 5 mM CaCl_2_. The protein eluted as two separate peaks (Fig. 3b and Additional file 4: Figure S3). Fractions corresponding to each oligomeric form were pooled separately, concentrated and aliquoted before flash-freezing in liquid nitrogen for storage at 193 K.

To determine the molecular weight of the two oligomeric forms of hS100A8 obtained during SEC (Additional file 4: Figure S3), an aliquot of the pooled hS100A8 sample obtained after the Source 15Q column was also run onto a 24 ml Superdex 75 column (GE Healthcare) equilibrated in 20 mM HEPES pH 7.5, 200 mM NaCl, 5 mM CaCl_2_ and connected to a BioLogic DuoFlow HPLC system (BioRad). Calibration of the SEC column was performed in the same buffer as for the S100A8 run using the Gel Filtration Markers Kit for Protein Molecular Weights 6500–66000 Da (Sigma-Aldrich).

### Crystallization of Zn^2+^/Ca^2+^-hS100A8

Crystallization of hS100A8 in the presence of zinc was performed with both the dimeric (10 mg ml^−1^) and the tetrameric (3.2 mg ml^−1^) forms of the protein after adding 3 mM ZnCl_2_ to the protein solution. Crystallization was carried out using the sitting-drop vapor-diffusion technique in 96-well Swissci MRC crystallization plates with the help of a MOSQUITO robot (TTP LabTech) and commercial screens from Hampton Research (INDEX screen) and Molecular Dimensions Ltd (Structure Screen, PROPLEX, MacroSol and Stura Footprint screens). Several hits were obtained for the dimeric form whereas only a few small crystals appeared for the tetramer, probably due to the much lower protein concentration imposed by precipitation at higher concentrations. Two conditions were further optimized by varying the precipitant concentration, pH, ionic strength, and protein concentration, and by screening with an additive collection (Hampton Research). The final optimal conditions were 0.2 M ammonium acetate, 0.1 M Na acetate pH 4.0, 15 % PEG 3350, 0.4 % 2,2,2-trifluoroethanol for crystal form 1 and 5 mM ZnCl_2_, 0.1 M Na cacodylate pH 6.5, 8 % PEG 8000 for crystal form 2.

### Data collection and structure determination

Before flash-cooling into liquid nitrogen, crystals were soaked a few seconds into a cryoprotective solution corresponding to the reservoir of their crystallization drop but containing 35 % PEG 3350 instead of 15 % (crystal form 1) or with 25 % glycerol added (crystal form 2). Diffraction data were collected at 100 K on the I911-3 beamline at MAX-lab (Lund, Sweden) (Table 1). For each crystal, three complete datasets were collected: a native dataset at 1.0 Å, a single-wavelength anomalous dispersion (SAD) dataset at the Zn peak (λ = 1.27 Å), and a third dataset after the Zn peak (λ = 1.30 Å). All datasets were processed and scaled using XDS [45].

Crystal form 1 displayed a P2_1_2_1_2_1_ symmetry with eight molecules of hS100A8 per asymmetric unit and diffracted to a maximal resolution of 2.2 Å. Crystal form 2 displayed a C222_1_ symmetry with four molecules of hS100A8 per asymmetric unit and the data could be processed to a maximum resolution of 2.1 Å. Both structures were solved by molecular replacement with PHASER [46] from the PHENIX package [37] using the structure of human S100A8 bound to calcium [14] as a search model. Refinement of the initial models was carried out by alternating cycles of manual rebuilding in COOT [47] and cycles of positional refinement with PHENIX.REFINE [37] using individual isotropic Atomic Displacement Parameters (ADP) as well as Translation–Libration–Screw (TLS) parameterization (Table 1). The final models were validated with Molprobity [48]. All figures were made with the Pymol Molecular Graphics System (version 0.99rc6, DeLano Scientific LLC).

### Ethics

Ethics was not requested for this study since it does not involved human participants or animals, and the human protein we crystallized was expressed recombinantly in a bacterial system from a synthetic DNA construct.

### Consent to publish

Not applicable.

### Availability of data and materials

The coordinates and structure factors for the structures of Zn^2+^/Ca^2+^-hS100A8 from crystal forms 1 and 2 have been deposited in the Protein Data Bank with accession numbers 5HLV and 5HLO, respectively. Additional files can be found on the online version of this paper and contain Additional file 1: Table S1, Additional file 2: Figure S1, Additional file 3: Figure S2 and Additional file 4: Figure S3. The legend for all the additional files is specified at this end of this article.

## Additional files

Additional file 1: Table S1. Data collection and processing statistics for the datasets collected at wavelengths of 1.27 Å and 1.30 Å for both crystal forms 1 and 2. Values for the outer shell are given in parentheses. All datasets were processed with XDS [45] with the Friedel pairs kept separated. (DOCX 24 kb) Additional file 2: Figure S1. Identification of the new Zn-sites in the Zn^2+^/Ca^2+^-hS100A8 structure derived from crystal form 2 (C222_1_). *(a)* Experimental map containing anomalous data obtained after SAD-phasing in PHENIX.AUTOSOLVE [37] displayed as magenta mesh and contoured at 3.5 σ. The final refined model is superimposed for comparison. 12 anomalous sites were identified, including the 8 Zn^2+^ sites and 4 of the 8 Ca^2+^ sites (Ca2, Ca3, Ca6 and Ca8). *(b)* Anomalous difference Fourier map calculated using phases and weight from the best refined atomic model (without ions) obtained with the native dataset (crystal form 2) and anomalous differences from the datasets collected at a wavelength of 1.27 Å (magenta mesh, contour at 3.5 σ). *(c)* Anomalous difference Fourier map calculated using phases and weight from the best refined atomic model (without ions) obtained with the native dataset (crystal form 2) and anomalous differences from the datasets collected at a wavelength of 1.30 Å (red mesh, contour at 3.5 σ). The anomalous signal disappears for all Zn^2+^ ions but remains for the Ca^2+^ ions. (PDF 1749 kb) Additional file 3: Figure S2. Close-up view on the His-Zn binding motif of several S100 proteins. *(a)* Crystallographic structure of hS100A7 in the presence of both zinc and calcium [31]. *(b)* Crystallographic structure of hS100A12 in the presence of zinc and in the absence of calcium [34]. *(c)* Crystallographic structure of hS100A15 in the presence of both zinc and calcium [33]. *(d)* Crystallographic structure of hS100B in the presence of both zinc and calcium [32]. (PDF 2784 kb) Additional file 4: Figure S3. Calibration of the Size Exclusion Chromatography (SEC) column and composition of the two oligomeric forms obtained by SEC for hS100A8. *(a)* Theoretical molecular weights and measured elution volumes of the different protein standards used for calibration of the 24 ml SEC column (Superdex 75, GE Healthcare Lifesciences). The calibration was performed in 20 mM HEPES pH 7.5, 200 mM NaCl and 5 mM CaCl_2_. *(b)* Calibration curve derived from the SEC runs with the different protein standards. *(c)* Elution profile of hS100A8 on the SEC column equilibrated in 20 mM HEPES pH 7.5, 200 mM NaCl and 5 mM CaCl_2_. In the sole presence of calcium, hS100A8 elutes as two peaks with elution volumes of 11.15 (Peak 1) and 12.80 (Peak 2), respectively. *(d)* Molecular weights for the two hS100A8 species and corresponding molecular composition extrapolated from the calibration curve. The equation used to calculate the molecular weights is indicated above the table. (PDF 107 kb)

## Abbreviations

DAMPs: damage-associated molecular patterns
hS100A8: human S100A8
NETs: neutrophil extracellular traps
r.m.s.d.: root mean square deviation
SEC: size exclusion chromatography
