# Supplementary material for: Crystal structure of human S100A8 in complex with zinc and calcium
Source: BMC Struct Biol. 2016 Jun 1;16:8. doi: 10.1186/s12900-016-0058-4 (PMC4888247; doi:10.1186/s12900-016-0058-4)
Supplement: Additional file 1: Table S1. — Data collection and processing statistics for the datasets collected at wavelengths of 1.27 Å and 1.30 Å for both crystal forms 1 and 2. Values for the outer shell are given in parentheses. All datasets were processed with XDS [45] with the Friedel pairs kept separated. (DOCX 24 kb) [file 12900_2016_58_MOESM1_ESM.docx]

|  | **Crystal form 1**  **λ = 1.27 Å** | **Crystal form 1**  **λ = 1.30 Å** | **Crystal form 2**  **λ = 1.27 Å** | **Crystal form 2**  **λ = 1.30 Å** |
| --- | --- | --- | --- | --- |
| Diffraction source | I911-3, MAX-lab | I911-3, MAX-lab | I911-3, MAX-lab | I911-3, MAX-lab |
| Wavelength (Å) | 1.27 | 1.30 | 1.27 | 1.30 |
| Temperature (K) | 100 | 100 | 100 | 100 |
| Detector | MARMOSAIC 225 mm CCD | MARMOSAIC 225 mm CCD | MARMOSAIC 225 mm CCD | MARMOSAIC 225 mm CCD |
| Crystal-to-detector distance (mm) | 314 | 314 | 148 | 148 |
| Rotation range per image (°) | 1 | 1 | 1 | 1 |
| Total rotation range (°) | 200 | 200 | 200 | 200 |
| Space group | P2_1_2_1_2_1_ | P2_1_2_1_2_1_ | C222_1_ | C222_1_ |
| *a*, *b*, *c* (Å) | 50.98, 85.15, 197.00 | 50.98, 85.15, 197.08 | 55.94, 89.85, 196.41 | 55.95, 89.90, 196.55 |
| α, β, γ (°) | 90, 90, 90 | 90, 90, 90 | 90, 90, 90 | 90, 90, 90 |
| Mosaicity (°) | 0.11 | 0.11 | 0.23 | 0.23 |
| Resolution range (Å) | 50 – 3.5 (3.6 – 3.5) | 50 – 3.5 (3.6 – 3.5) | 50 – 2.1 (2.2 – 2.1) | 50 – 2.1 (2.2 – 2.1) |
| Total No. of reflections | 82214 | 77474 | 230140 | 232105 |
| No. of unique reflections | 20524 | 19740 | 54592 | 54589 |
| Completeness (%) | 98.2 (81.7) | 94.4 (44.2) | 97.8 (96.3) | 97.7 (96.2) |
| Redundancy | 4.0 (1.9) | 3.9 (1.2) | 4.2 (4.2) | 4.2 (4.2) |
| *I* / σ(*I)* | 12.10 (4.38) | 15.77 (5.28) | 14.29 (2.07) | 14.26 (1.52) |
| *R*_meas_ | 12.8 (23.8) | 9.3 (13.7) | 8.8 (87.7) | 9.8 (118.5) |
| SigAno (\|F(+)-F(-)\|/Sigma) |  |  |  |  |
| Inner shell (50 – 20 Å) | 1.709 | 0.938 | 3.664 | 1.905 |
| Outer shell | 0.832 | 0.869 | 0.756 | 0.717 |
| Overall | 0.988 | 0.836 | 1.250 | 0.860 |
| Wilson B factor (Å^2^) | 18.1 | 17.1 | 35.4 | 40.0 |
