# Supplementary figures and images for: Crystal structure of human S100A8 in complex with zinc and calcium
Source: BMC Struct Biol. 2016 Jun 1;16:8. doi: 10.1186/s12900-016-0058-4 (PMC4888247; doi:10.1186/s12900-016-0058-4)

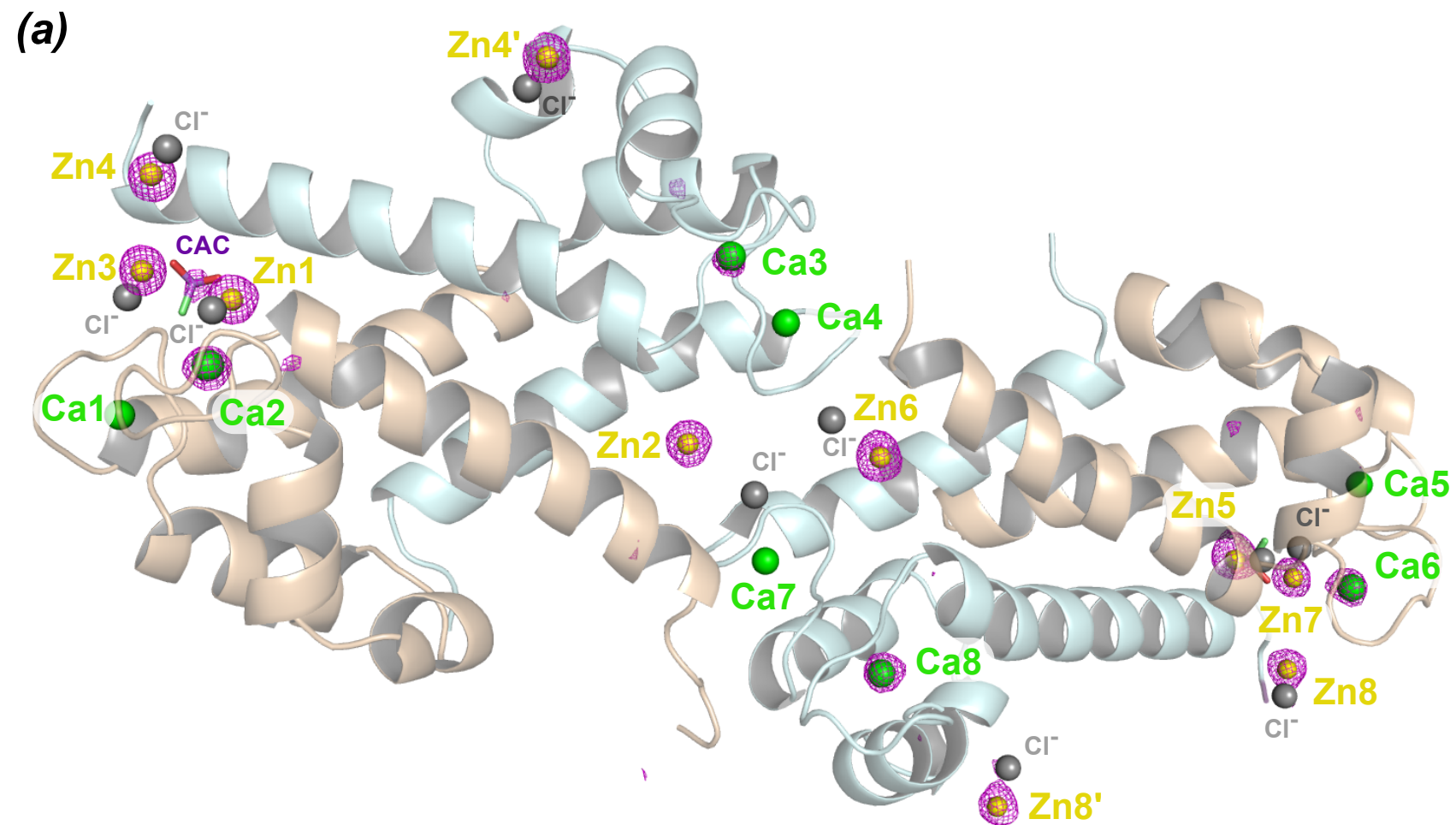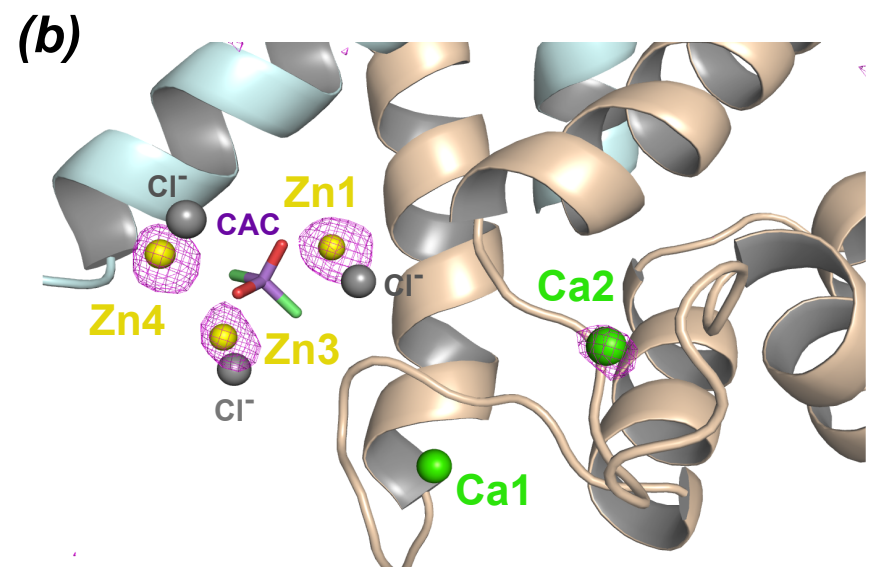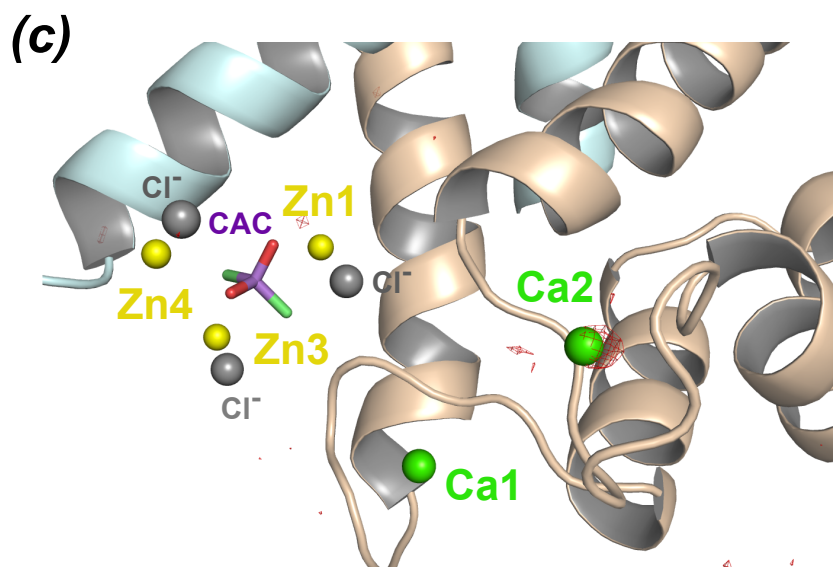

Supplement: Additional file 2: Figure S1. — Identification of the new Zn-sites in the Zn2+/Ca2+-hS100A8 structure derived from crystal form 2 (C2221). (a) Experimental map containing anomalous data obtained after SAD-phasing in PHENIX.AUTOSOLVE [37] displayed as magenta mesh and contoured at 3.5 σ. The final refined model is superimposed for comparison. 12 anomalous sites were identified, including the 8 Zn2+ sites and 4 of the 8 Ca2+ sites (Ca2, Ca3, Ca6 and Ca8). (b) Anomalous difference Fourier map calculated using phases and weight from the best refined atomic model (without ions) obtained with the native dataset (crystal form 2) and anomalous differences from the datasets collected at a wavelength of 1.27 Å (magenta mesh, contour at 3.5 σ). (c) Anomalous difference Fourier map calculated using phases and weight from the best refined atomic model (without ions) obtained with the native dataset (crystal form 2) and anomalous differences from the datasets collected at a wavelength of 1.30 Å (red mesh, contour at 3.5 σ). The anomalous signal disappears for all Zn2+ ions but remains for the Ca2+ ions. (PDF 1749 kb) [file 12900_2016_58_MOESM2_ESM.pdf]

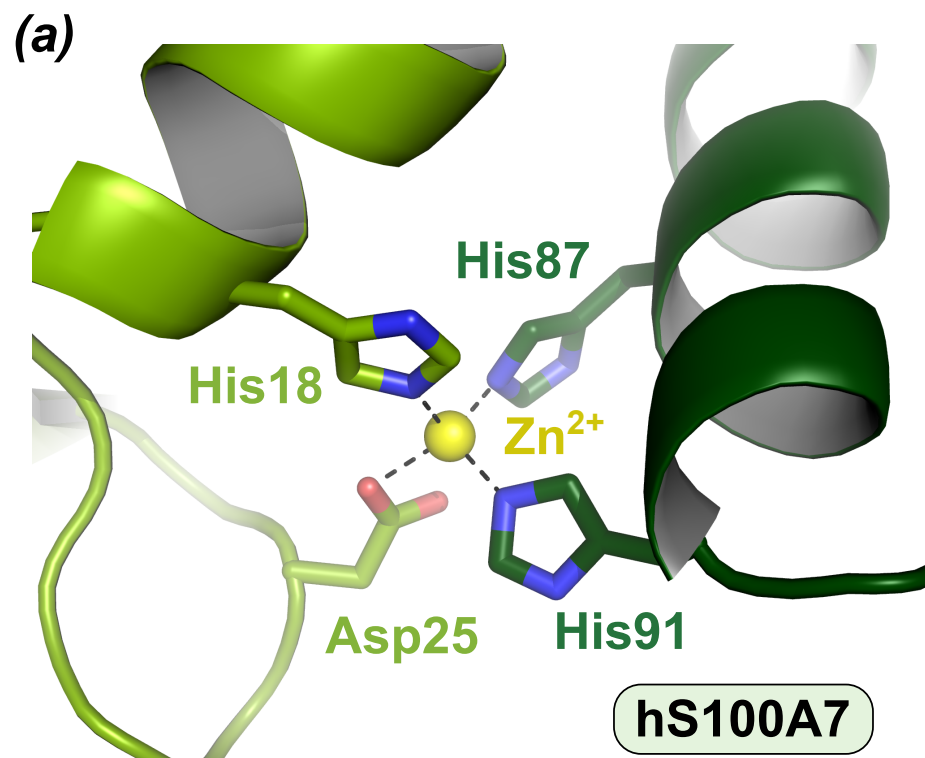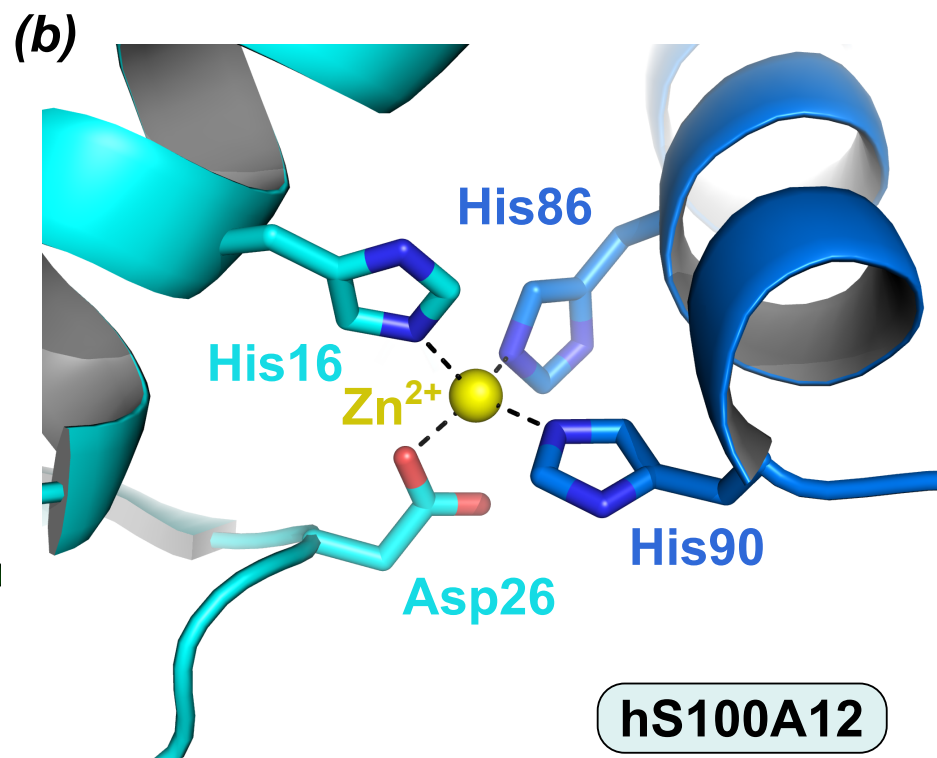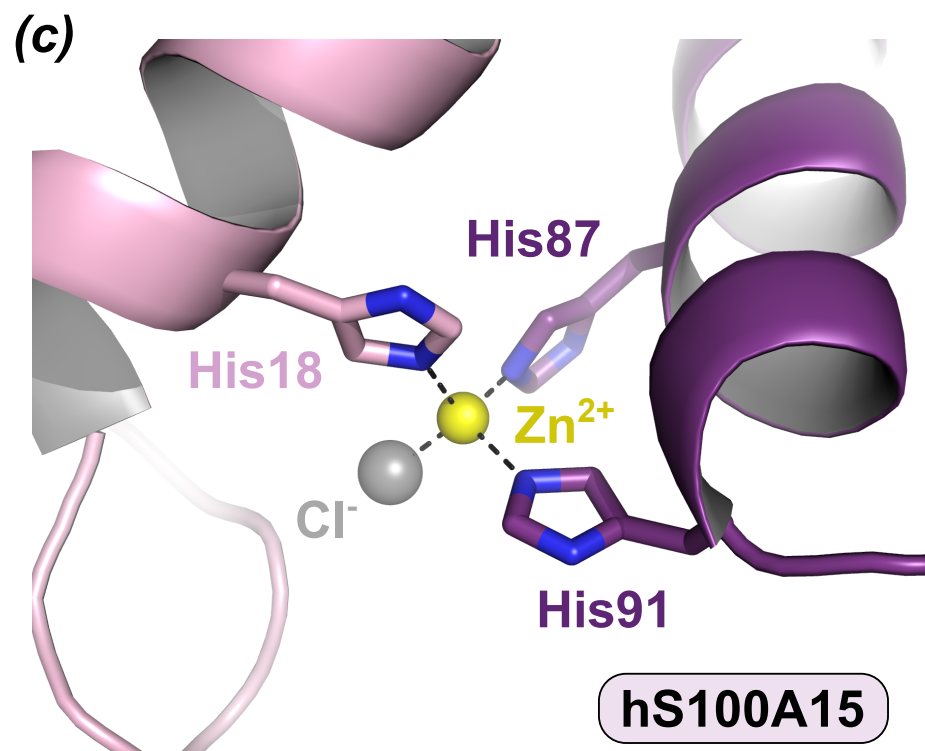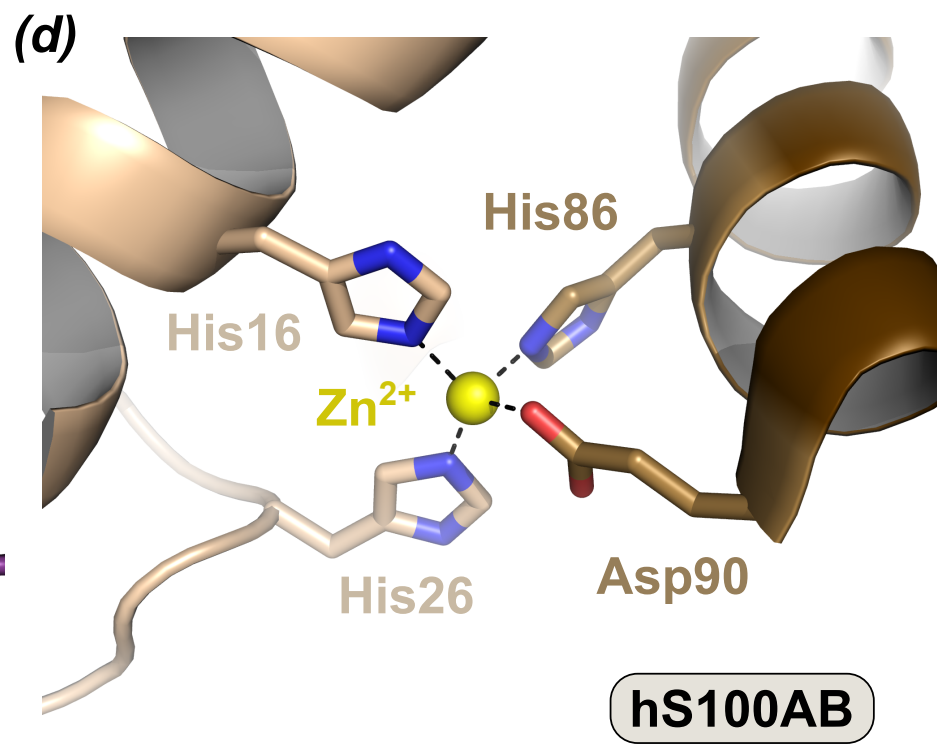

Supplement: Additional file 3: Figure S2. — Close-up view on the His-Zn binding motif of several S100 proteins. (a) Crystallographic structure of hS100A7 in the presence of both zinc and calcium [31]. (b) Crystallographic structure of hS100A12 in the presence of zinc and in the absence of calcium [34]. (c) Crystallographic structure of hS100A15 in the presence of both zinc and calcium [33]. (d) Crystallographic structure of hS100B in the presence of both zinc and calcium [32]. (PDF 2784 kb) [file 12900_2016_58_MOESM3_ESM.pdf]
