# Supplementary material for: Crystal structure of human S100A8 in complex with zinc and calcium
Source: BMC Struct Biol. 2016 Jun 1;16:8. doi: 10.1186/s12900-016-0058-4 (PMC4888247; doi:10.1186/s12900-016-0058-4)
Supplement: Additional file 4: Figure S3. — Calibration of the Size Exclusion Chromatography (SEC) column and composition of the two oligomeric forms obtained by SEC for hS100A8. (a) Theoretical molecular weights and measured elution volumes of the different protein standards used for calibration of the 24 ml SEC column (Superdex 75, GE Healthcare Lifesciences). The calibration was performed in 20 mM HEPES pH 7.5, 200 mM NaCl and 5 mM CaCl2. (b) Calibration curve derived from the SEC runs with the different protein standards. (c) Elution profile of hS100A8 on the SEC column equilibrated in 20 mM HEPES pH 7.5, 200 mM NaCl and 5 mM CaCl2. In the sole presence of calcium, hS100A8 elutes as two peaks with elution volumes of 11.15 (Peak 1) and 12.80 (Peak 2), respectively. (d) Molecular weights for the two hS100A8 species and corresponding molecular composition extrapolated from the calibration curve. The equation used to calculate the molecular weights is indicated above the table. (PDF 107 kb) [file 12900_2016_58_MOESM4_ESM.pdf]

(a)

Protein standard	Molecular Weight (M_W) in Da	Elution Volume V_E (in ml)
Blue Dextran	V_O	8.91
BSA	66500	10.77
Carbonic Anhydrase	29000	12.75
Cytochrome c	12400	14.37
Aprotinin	6500	16.30

V_C = 24 ml

(b)

$$K_{AV} = \frac{V_E - V_O}{V_C - V_O}$$

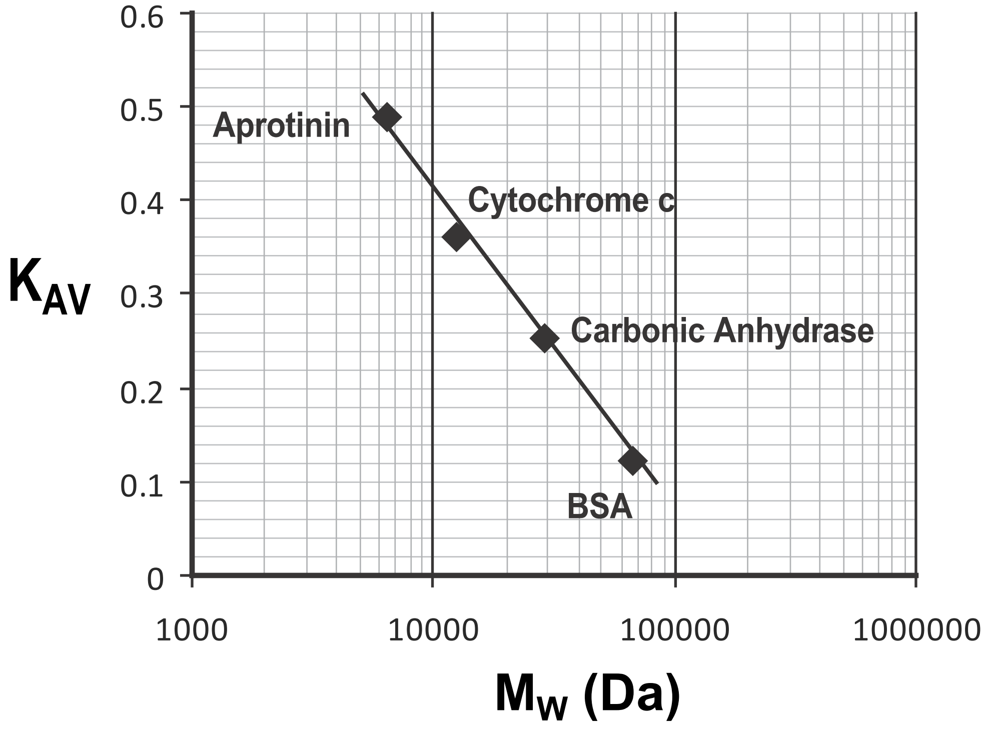

(c)

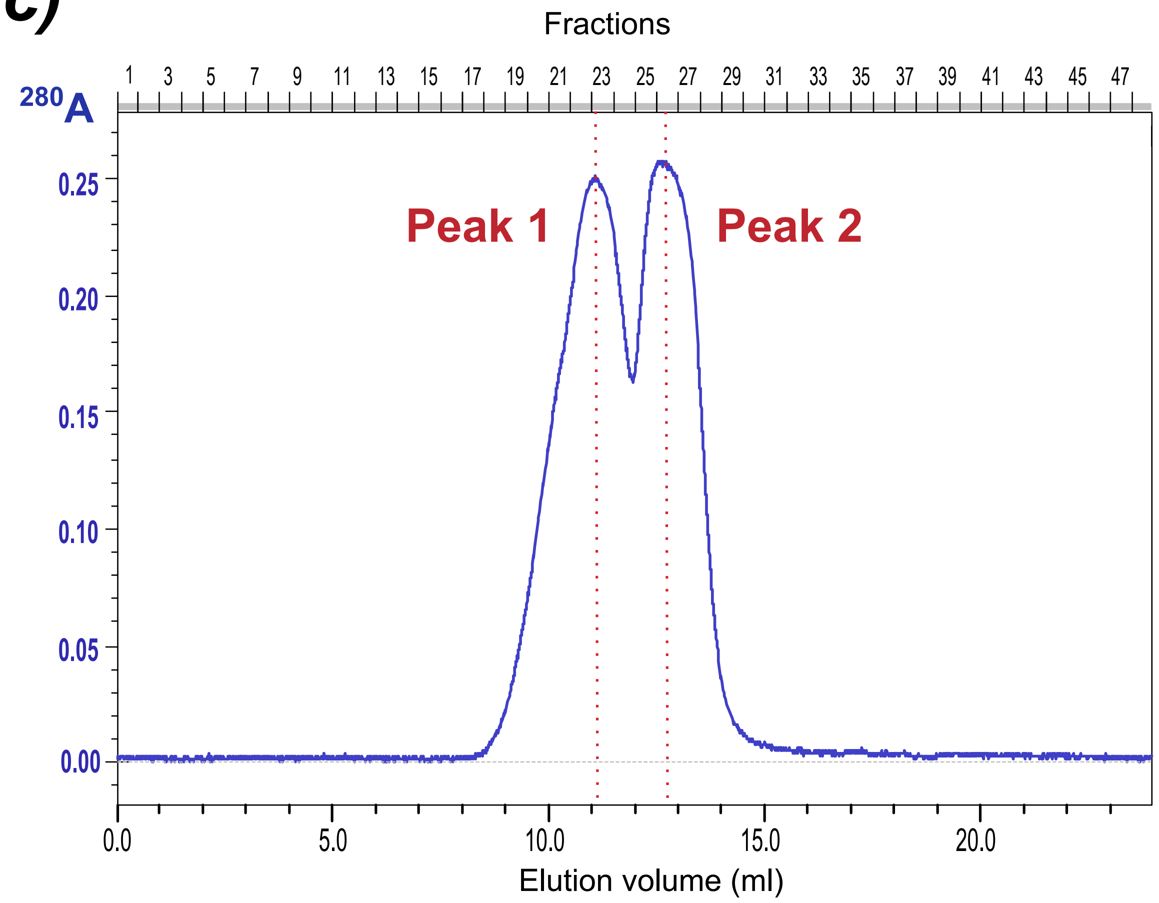

(d)

$$K_{AV} = -0.3538 * \log(M_W) + 1.8276$$

hS100A8 species from SEC	Measured Elution Volume V_E (in ml)	Extrapolated Molecular Weight (M_W) in Da	Corresponding number of hS100A8 dimers
Peak 1	11.15	55726	2.55
Peak 2	12.80	27353	1.25
